# Lockdown Effects on Healthy Cognitive Aging During the COVID-19 Pandemic: A Longitudinal Study

**DOI:** 10.3389/fpsyg.2021.685180

**Published:** 2021-05-24

**Authors:** Martina Amanzio, Nicola Canessa, Massimo Bartoli, Giuseppina Elena Cipriani, Sara Palermo, Stefano F. Cappa

**Affiliations:** ^1^Department of Psychology, University of Turin, Turin, Italy; ^2^European Innovation Partnership on Active and Healthy Ageing, Brussels, Belgium; ^3^Department of Humanities and Life Sciences, Scuola Universitaria Superiore Istituto Universitario di Studi Superiori (IUSS), Pavia, Italy; ^4^Istituti Clinici Scientifici Maugeri IRCCS, Cognitive Neuroscience Laboratory of Pavia Institute, Pavia, Italy; ^5^UOC Neuroradiologia -IRCCS Istituto Neurologico Carlo Besta, Milan, Italy; ^6^Istituto di Ricovero e Cura a Carattere Scientifico (IRCCS) Mondino Foundation, Pavia, Italy

**Keywords:** normal aging, executive functions, mood deflections, gait speed, handgrip strength, lockdown pandemic fatigue

## Abstract

The COVID-19 pandemic is a health issue leading older adults to an increased vulnerability to unfavorable outcomes. Indeed, the presence of physical frailty has recently led to higher mortality due to SARS-CoV-2 infection. However, no longitudinal studies have investigated the role of neuropsychogeriatric factors associated with lockdown fatigue in healthy cognitive aging. Eighty-one healthy older adults were evaluated for their neuropsychological characteristics, including physical frailty, before the pandemic (T0). Subsequently, 50 of them agreed to be interviewed and neuropsychologically re-assessed during the lockdown (T1) and immediately after it (T2). Moreover, during another home confinement, they performed a psychological screening (T3) to evaluate possible mood changes and fatigue. According to Fried's frailty criteria, at T0, 63% of the sample was robust, 34.5% pre-frail, and only 2.5% frail. Significantly, these subjects presented a decrease in handgrip strength and walking speed (29.6 and 6.1%, respectively). Results from Principal Component Analyses and multiple regression models highlighted the contribution of “cognitive” and “psychological” factors (i.e., attentive-executive performance and mood deflections) in explaining handgrip strength and gait speed. At T3, lockdown fatigue was explained by higher scores on the Beck Depression Inventory and lower scores on the Trail Making Test part A. Results from a moderated-mediation model showed that the effect of psychomotor speed on lockdown fatigue was mediated by depression, with a moderating effect of gait speed. Our findings highlight the complex interrelationship between cognitive, psychological, and physical factors in the emergence of pandemic fatigue in a carefully selected older population.

## Introduction

The presence of a state of frailty, characterized by a clinical history of polypathology, has recently led to higher mortality due to the SARS-CoV-2 pandemic among the older population (Onder et al., 2020). Polypathological subjects suffer from two or more chronic diseases, which could lead to disability and higher mortality rates (Gómez-Salgado et al., 2019).

The increased susceptibility to epidemic effects is likely to be determined not only by existing comorbidity conditions, but also by reduced immunity, partly due to the physiological aging process (Gavazzi and Krause, 2002).

Lockdown measures play a major role in the containment of SARS-CoV-2 infection. However, isolation and social distancing are associated with cognitive decline, depression, and anxiety in the older population (Santini et al., 2020). Faced with prolonged confinement, older people may experience loneliness, pessimism, health problems, negative stereotyping, and sleep disorders (Luchetti et al., 2020). Furthermore, social isolation is an important public health issue that can lead to higher probabilities of cognitive and mental problems (Gerst-Emerson and Jayawardhana, 2015).

Recently, a feeling of “lockdown fatigue” has been associated with home confinement due to the COVID-19 pandemic. Only a few authors have investigated this particular phenomenon, without specifically targeting the older population.

For instance, Bartoszek et al. (2020) examined a sample of 471 subjects predominantly young (mean age = 25 years, SD = 2.1) and female (85.6%). The authors found that living alone and starting new therapies during the lockdown were associated with higher levels of fatigue. In a cross-sectional community-based survey, COVID-19 pandemic fatigue was experienced in about 64% of the sample; again, the over-60 population was underrepresented (Morgul et al., 2020). Labrague and Ballad (2020) analyzed the fatigue among Philippines college students during the COVID-19 lockdown in a remote cross-sectional study. Their results indicated moderate levels of fatigue, in terms of physical exhaustion, body pain, headaches, worries, and reduced motivation. Finally, in a survey of 260 subjects (mean age = 47 years), lockdown fatigue was related to worsened mood and lifestyle (Field et al., 2021).

Despite the problems highlighted in these studies, to date no longitudinal ones have analyzed the associations among lockdown fatigue, physical conditions, cognitive functions, and mood deflections in cognitively normal older adults during home confinement.

This is unfortunate, as targeted psychological interventions may have a role in primary prevention and psychological well-being, by enhancing resilience during periods of health emergency while also reducing the negative impact on physical and cognitive dysfunctions and mood deflections disorders.

To fill this gap, we investigated the relationships between pandemic lockdown fatigue, physical-cognitive functions, and mood changes in 50 older adults−60 years and older (World Health Organization, 2021)—engaged as volunteers, suffering from two or more age-related diseases (polypathology) and receiving subsequent pharmacotherapy (Masnoon et al., 2017).

The availability of pre-COVID19 baseline data (T0) of this sample allowed us to investigate the contribution of cognitive functioning and psychological state in explaining the variability of grip strength and gait speed, two crucial components of the frailty phenotypic model (Fried et al., 2001).

A longitudinal approach (from April 2019 to January 2021) was used to analyze: (a) the neuropsychogeriatric profile of the participants before and after the lockdown; (b) whether these subjects had developed lockdown pandemic fatigue; (c) whether this fatigue could be predicted by variables measured at baseline using a moderate-mediation model.

In particular, as lockdown fatigue had been previously related to depressive symptoms (Bartoszek et al., 2020; Field et al., 2021; Seiter and Curran, 2021) and a slowdown in attentional abilities (Fiorenzato et al., 2021), we expected both to be useful in predicting exhaustion due to the restrictive measures in our sample. Furthermore, as mood deflection may be influenced by cognitive dysfunction (e.g., attention deficit; Keller et al., 2019), and physical frailty (Buigues et al., 2015)—particularly gait speed (Marino et al., 2019)—we hypothesized that attentional skills and gait speed would interact in modulating depression mood changes.

Our hypothesis is that decreased psychomotor and gait speed may interact to produce a depressive state that mediates their effect on lockdown fatigue.

## Materials and Methods

### Participants

The creation of an innovative laboratory (LAB) on active and healthy aging for training, education, research, and development allowed neuropsychological interventions on members of the University of the Third Age in Turin (UNITRE-TO). The subjects attending the LAB activities were numerous (*N* = 200) and, during the academic year 2018–19, they followed specific teaching modules aimed at learning functional lifestyles in view of active and healthy aging.

Eighty-one Italian volunteer subjects (64 females, age range 60–84) out of the 200 participants (40.5%) were selected if they: (a) suffered from at least two chronic pathologies (see Supplementary Table 1) and received pharmacotherapy treatments (Smith and Bondi, 2013); (b) were aged 60 years or older in order to be classified as “older adults” (World Health Organization, 2021); and (c) had a Mini Mental State Examination (MMSE, Folstein et al., 1975) raw score ≥ 27. The latter is the best cut-off for Mild Cognitive Impairment (MCI) detection in highly educated populations (O'Bryant et al., 2008), such as the one involved in our study. Moreover, the subjects did not report subjective cognitive decline (Jessen et al., 2020) and had not undergone any medical or neurological examination for suspected MCI (Petersen and Negash, 2008).

Participants were excluded from the study if they: (1) suffered from psychiatric or neurocognitive disorders based on DSM-5 criteria (American Psychiatric Association, 2013); (2) were taking any medications that could substantially affect cognitive functioning.

All subjects were functionally independent and socially active. We performed a screening in order to investigate their cognitive, functional, and behavioral domains, and to detect the mildest neuropsychogeriatric dysfunction considering their physical health status, their cognitive functioning, and possible mood deflections in terms of anxiety and depression (T0).

During the COVID-19 pandemic, 50 out of the 81 subjects agreed to participate in LAB activities remotely in compliance with restrictive lockdown measures. They underwent semi-structured interviews (T1), neuropsychological (T2), and psychological assessment (T3) to identify any possible under cut-off values in their individual profile.

All participants gave written informed consent prior to the study, which was performed in accordance with the Declaration of Helsinki and approved by the Ethics Committee of the University of Turin (Prot. n. 10038 and Prot. n. 151786, before and after the pandemic, respectively).

### Assessments

#### Assessments Measures at the Baseline (T0)

From April to October 2019, the subjects underwent a multidimensional neuropsychogeriatric assessment, which consisted of cognitive tests, functional, and behavioral scales. Three different neuropsychologists evaluated the subjects in two experimental sessions, held 1 day apart and each lasting about 90 min, in order to prevent fatigue and lack of adherence to the tasks.

Information about clinical history, symptoms, and chronic pathologies was collected using the Cumulative Illness Rating Scale (CIRS: Linn et al., 1968).

Global cognitive functioning was assessed by using the Addenbrooke's Cognitive Examination—Revised version (ACE-R: Mioshi et al., 2006) and the Montreal Cognitive Assessment (MoCA: Conti et al., 2015). Then, a detailed evaluation was carried out considering several cognitive domains: episodic long-term memory (Rey Memory Test and Short Story Recall: Spinnler and Tognoni, 1987), short-term memory (Digit Span and Corsi Test: Spinnler and Tognoni, 1987), attention (attentional matrices: Spinnler and Tognoni, 1987), language and fluencies (Token Test—TT: De Renzi and Vignolo, 1962; Phonemic and Semantic Fluency: Spinnler and Tognoni, 1987; Naming subtest of Aachner Aphasia Test—AAT: De Bleser et al., 1986), visuo-constructive abilities (Copy Design: Carlesimo et al., 1996), problem-solving (Raven's Coloured Progressive Matrices—CPM-36: Spinnler and Tognoni, 1987), and executive functions (Trail Making Test—TMT: Giovagnoli et al., 1996).

Psychiatric rating scales were used to assess: depression (Beck Depression Inventory—BDI: Beck et al., 1988), apathy (Apathy Evaluation Scale—AES: Marin, 1996), anxiety (Hamilton Rating Scale for Anxiety—HAM-A: Hamilton, 1959), disinhibition (Disinhibition Scale—DIS-S: Starkstein et al., 2004), and hypomania (Mania Scale—MAS: Bech et al., 1978).

The possible presence of a physical frailty status was assessed by adopting the phenotypic model (Fried et al., 2001). According to Fried's criteria, frailty is a pathophysiological syndrome characterized by 5 determinants: (1) unintentional weight loss; (2) limitation of physical activity; (3) asthenia; (4) handgrip strength reduction; and (5) slowing in walking speed. The presence of three out of five criteria indicates a state of frailty, while one or two criteria identify a pre-frailty status.

#### Semi-Structured Interviews at T1

From April to May 2020, during the lockdown period, all 81 subjects assessed at T0 were contacted by phone, text message, or email in order to ask for their availability to participate in the study. Fifty of them agreed.

At first, a semi-structured interview was carried out to collect information about housing status, health conditions, measures to avoid contagion, and sources of information considered as reliable. In addition, they were asked for any changes in their habits in terms of diet, physical activity, sleep, smoking, use of drugs and alcohol, and cognitively stimulating activities (Supplementary Table 2).

At the end of the interview, the participants were asked to schedule a second appointment for a neuropsychological evaluation.

#### Neuropsychological Assessment at T2

This third phase was carried out remotely via video-call from May to July 2020, during the so-called “phase 2” of the Italian quarantine, when restrictive measures were eased.

The choice of neuropsychological tests was as close as possible to T0. Cognitive functioning was assessed by ACE-R, and MMSE. The test subsections involving visuo-spatial skills were presented in screen-sharing mode. We also used the MoCA-Blind test (Wittich et al., 2010). This version was originally designed for people with visual impairment. In our research, we used such instrument in order to overcome the issues related to the performance of some of the visual tasks of the original test (e.g., TMT-B, short version) through the screen-sharing mode. The blind version is scored out of 22 but, as suggested on www.mocatest.org (Nasreddine, 2020), the official MoCA website, it needs to be converted back to 30 as the original test.

Mood changes were evaluated using BDI, AES, HAM-A, DIS-S, and MAS. The socioeconomic characteristics of the sample were analyzed using the Four Factor Index of Social Status (Hollingshead index, HI: Hollingshead, 1975, 2011). This index is based on the educational level and the type of employment of family members (for more details on scoring, please see Hollingshead, 1975, 2011). In this case, since our group consisted of retired people, their last job was taken into account.

#### Neuropsychological Assessment at T3

Remote phone calls were used to carry out this last phase, which occurred during another period of very restrictive measures in Italy (December 2020–January 2021).

In order to achieve our aims, we chose to test the subjects only on BDI and HAM-A. These scales are very suitable for a remote assessment and, furthermore, depression and anxiety are two of the most frequently reported and studied psychological aspects in the literature concerning the COVID-19 pandemic (Salari et al., 2020) and subsequent home confinement (Bartoszek et al., 2020; Morgul et al., 2020; Field et al., 2021).

In addition, we investigated the subjects' fatigue related to restrictive measures using the Lockdown Fatigue Scale (LFS: Labrague and Ballad, 2020), which has been recently designed to assess exhaustion during quarantine due to the COVID-19 pandemic. The LFS consists of 10 items concerning the psychological effects of lockdown, such as mood deflection, attentional deficits, and possible somatization (i.e., weakness, headache). Subjects are asked how often they experience those effects during home confinement. Each item is scored on a Likert scale that ranges from 1 (never) to 5 (always). This instrument allows the detection of four different levels of fatigue: low (1–12), mild (13–25), moderate (26–37), and severe (38–50).

### Data Analyses

#### Principal Component Analyses

T0 data were examined in order to investigate whether handgrip strength and walking speed could be predicted by a combination of variables concerning cognitive performance, psychological status, and physical comorbidity (Supplementary Table 1). In our analyses, only grip strength and walking speed were taken into consideration because: (a) their values presented more variance, not being dichotomous (*presence/absence*); and (b) they are more related to cognitive functioning—particularly concerning executive control (Hooghiemstra et al., 2017)—and mood changes (Gordon et al., 2019; Brooks et al., 2020).

To this purpose, in a preliminary data-reduction stage, two Principal Component Analyses (PCA) were used to unveil superordinate factors transcending: (a) the single scores of cognitive functioning; and (b) the single scales of psychological status. The first PCA was carried out on cognitive tasks, while the second included the scales used to assess possible mood changes in terms of depression (BDI), apathy (AES), anxiety (HAM-A), disinhibition (DIS-S), and hypomania (MAS). We retained the components with eigenvalue >0.70 (Jolliffe, 1972), using an orthogonal rotation (Varimax) to facilitate their interpretation. Subsequently, two multiple regression models were used to estimate the contribution of the resulting “cognitive” and “psychological” factors (independent variables), in explaining variability in grip strength and gait speed (adjusted for gender/BMI and for gender/height, respectively). Then, the “cognitive” and “psychological” factors associated with a significant effect were modeled in additional multiple regressions including grip strength and gait speed as dependent variables, and comorbidity, age, and education as predictors.

Based on the outcome of these analyses, we assessed a mediation-moderate model to investigate the direction of the relationship among the factors predicting lockdown pandemic fatigue. Specifically, a moderate mediation, also known as *conditional indirect effects*, occurs when the effect of the independent variable “TMT-A” on the outcome variable “LFS” via the mediating variable “BDI” differs according to the levels of the moderating variable “gait speed.”

#### Moderate-Mediation Model

We tested a moderate-mediation model to assess the hypothesis that: (a) depression at T0 mediates the negative relationship between attentional/executive resources (as tracked by TMT-A performance) at T0 and lockdown-fatigue scale at T3; and (b) this indirect effect is conditional on a moderating variable represented by frailty (as tracked by walking speed). Age, gender, and educational level were modeled as nuisance variables. We used the PROCESS macro (v.3.5) for SPSS (IBM, v.23) to test Hayes's (2017) model 7 (moderated mediation), after checking for the assumptions concerning the lack of outliers (based on Mahalanobis distance), the normal distribution of the residuals (Lilliefors, *p* > 0.05), multicollinearity (maximum variance inflation factor = 1.31; minimum tolerance = 0.763), independence of residuals (Durbin-Watson = 2.184), homoscedasticity (Breusch-Pagan test, *p* > 0.05). Our hypothesis concerning the moderation of mediated effects was tested through conditional process analysis based on Ordinary Least Squares (OLS) regression, using bootstrapping resampling (50,000 samples) to generate confidence intervals for direct and mediated effects, as well as for an index of moderated-mediation. Interaction variables were centered (to a mean of 0) before entering the analyses, and the Johnson and Neyman's (1936) approach was used to compute the range of significance and simple slopes for the interaction analyses, which were assessed 1 standard deviation below and above the mean. The statistical threshold was set at *p* < 0.05 (two-tailed).

## Results

### T0 Main Results

The mean scores obtained at T0 on global cognitive tests and behavioral scales are reported in Table 1. Even if the MMSE scores did not suggest the presence of MCI (i.e., O'Bryant et al., 2008) and the participants did not report subjective cognitive decline, their performance was below the reference cut-off value on some tests. In particular, on: MoCA (2.5%), Attentional matrices (1%), Copy design with programming elements (1%), Rey memory test instant recall (1%), Delayed recall (4%); Short story recall (1%), Digit span (4%); Corsi test (1%), and the Phonetic fluency test (4%). Although some deficits were found on neuropsychological tests, the percentages of under cut-off scores are consistent with the margin of error on tests in the normative population.

**Table 1:** Demographic and neuropsychological characteristics (T0).

	***N***	**M**	**SD**	**Cut-off**
**Demographic characteristics**				
Subjects	81			
Gender [male/female]	17/64			
Age [years]		70.15	6.37	
Education [years]		12.41	3.05	
**Neuropsychological assessment**				
Mini mental state examination		28.95	1.05	≥23.8
Addenbrooke's cognitive examination—revised version		91.68	4.89	≥79 (<75 years old)
				≥60 (>75 years old)
Montreal cognitive assessment		24.21	2.87	≥17.363
Rey memory test−15 instant words		42.16	9.11	≥ 28.53
Rey memory test−15 delayed words		8.54	2.95	≥ 4.69
Babcock short story recall test		16.93	4.45	≥ 8
Digit span forward		6.07	1.12	≥ 4.25
Corsi test		5.07	0.97	≥ 3.75
Phonemic verbal fluency		39.57	10.89	≥ 17.35
Semantic verbal fluency		25.00	5.27	≥ 7.25
Token test		32.67	2.56	≤ 29
Aachener aphasie test		115.63	2.56	≥ 106
Coloured progressive matrices-36		30.81	4.69	≥ 18.96
Attentional matrices		48.19	6.85	≥ 30
Coping design—without programming elements		10.32	1.25	≥ 7.18
Coping design—with programming elements		68.11	2.02	≥ 61.85
Trail making test-part A		42.36	12.73	≤ 94
Trail making test-part B		105.49	40.81	≤ 283
Trail making test B-A		63.14	34.90	≤ 187
**Neuropsychiatric assessment**				
Apathy evaluation scale		3.00	3.66	≤ 14
Beck depression inventory		8.53	5.63	≤ 9
Disinhibition scale		2.91	2.83	≤ 16.9
Hamilton rating scale for anxiety		5.83	4.44	≤ 14
Mania scale		2.40	2.65	≤ 15
**Functional assessment**				
CIRS—severity index		1.29	0.20	
CIRS—comorbidity index		0.93	0.91	

*N, number; M, mean; SD, standard deviation; CIRS, Cumulative Illness rating Scale*.

*Wherever there is a normative value, the cut-off scores are given in the statistical normal direction. For CIRS higher scores indicates greater coexistence of polypathologies and disease severity*.

Moreover, considering the behavioral scales, the subjects presented mood changes in terms of depression (BDI = 39%) and states of anxiety (HAM-A = 4%).

According to Fried criteria, 63% of the sample was robust, 34.5% pre-frail, and only 2.5% frail. Significantly, these subjects presented a decrease in handgrip strength and walking speed (29.6 and 6.1%, respectively).

As detailed above, a PCA led to reduce the initial dataset of cognitive scores (10 cognitive tests) to 5 factors explaining 82.8% of their variance (Table 2): (1) *Attention/Executive*, which refers to attention and executive functions, in term of cognitive set-shifting, assessed by the attentional matrices, and TMT-A, TMT-B; (2) *Memory*, represented by Short Story Recall (immediate and delayed recall); (3) *Visual-Constructive*, which refers to visuo-constructive abilities and abstract-non-verbal reasoning, assessed by Design Copying (with and without programming elements) and CPM-36; (4) *Language*, represented by AAT; (5) *Fluency*, represented by the Phonemic Verbal Fluency.

**Table 2:** Pattern matrix for the principal components analysis—cognitive factors (T0).

**Cognitive factors**	**Factor**				
	**1**	**2**	**3**	**4**	**5**
**Attention**					
Attentional matrices	**0.768**	0.311	0.231	0.226	−0.152
Trail making test-A	**0.873**	0.009	0.085	−0.067	0.294
Trail making test-B	**0.559**	0.492	0.104	0.097	0.443
**Language**					
Aachner aphasia test	0.071	0.133	0.073	**0.960**	0.158
**Memory**					
Short story recall—instant recall	0.030	**0.915**	0.196	0.051	−0.009
Short story recall—delayed recall	0.239	**0.880**	0.126	0.135	0.126
**Problem solving**					
Coloured progressive matrices-36	0.376	0.406	**0.541**	0.069	0.372
**Visuospatial abilities**					
Copy design—without programming elements	0.374	0.026	**0.716**	0.138	0.183
Copy design—with programming elements	−0.031	0.219	**0.838**	−0.006	0.031
**Verbal fluency**					
Phonemic fluency	0.124	0.057	0.162	0.156	**0.904**

*Factor loadings > 0.50 are expressed in bold type*.

We used the same approach to reduce the 5 scales of psychological status to 2 factors explaining 74.3% of their variance (Table 3): (1) *Mania-disinhibition*, measured with MAS, DIS-S; and (2) *Depression-apathy-anxiety*, assessed by BDI, HAM-A, AES (Table 2).

**Table 3:** Pattern matrix for the principal components analysis—psychological factors (T0).

**Psychological factors**	**Factor**	
	**1**	**2**
Beck depression inventory	−0.102	**0.790**
Hamilton rating scale for anxiety	0.418	0.698
Mania scale	**0.946**	−0.128
Apathy evaluation scale	0.093	**0.774**
Disinhibition scale	**0.890**	0.319

*Factor loadings > 0.50 are expressed in bold type*.

Subsequently, the contribution of these components was estimated in order to explain the variability of the crucial determinants of Fried's phenotypic model. When testing the predictors of grip strength, a significant model (*p* = 0.0051) showed that “cognitive” and “psychological” status explained 13% of the variance. In particular, increased grip strength reflected: (a) an increase of the Attention/Executive performance FACTOR 1-on PCA 1 (*p* = 0.0228); and (b) a decrease of “depression-apathy-anxiety,” FACTOR 2-on PCA 2 (*p* = 0.01006). There was no multicollinearity among explanatory variables (maximum variance inflation factor: *VIF* = 1.004), and the residuals were normally distributed (Kolmogorov–Smirnov test: *K-S* = 0.7795, *p* > 0.2). Moreover, 15% of the variance in gait speed (*p* = 0.0015) was also explained by Attention/Executive performance FACTOR 1-on PCA 1 (*p* = 0.0046), and depression-apathy-anxiety FACTOR 2-on PCA 2 (*p* = 0.0100). There was no multicollinearity among explanatory variables (maximum *VIF* = 1.004), and the residuals were normally distributed (*K-S* = 0.05775, *p* > 0.2). None of the other cognitive or psychological factors was significantly associated with handgrip strength or walking speed. To refine this finding, the contribution of Attention/Executive and depression-apathy-anxiety factors to grip strength and gait speed was assessed while taking into account age, education, and comorbidity (in terms of CIRS). In line with the above results, a significant model showed that 10% of the variance in grip strength (*r*^2^ = 0.10395, *p* = 0.0051) was explained by the Attention/Executive performance FACTOR 1-on PCA 1 (*p* = 0.0229), and depression-apathy-anxiety FACTOR 2-on PCA 2 (*p* = 0.0100). There was no multicollinearity among explanatory variables (maximum *VIF* = 1.37), and the residuals were normally distributed (*K-S* = 0.07795, *p* > 0.2). Moreover, a significant model showed that 13% of the variance in gait speed (*r*^2^ = 0.1315, *p* = 0.0015) was explained by the same factors: Attention/Executive performance FACTOR 1-on PCA 1 (*p* = 0.0046) and depression-apathy-anxiety FACTOR 2-on PCA 2 (*p* = 0.0100). There was no multicollinearity among explanatory variables (maximum *VIF* = 1.37), and the residuals were normally distributed (*K-S* = 0.05774, *p* > 0.2).

### T1 Main Results

The subjects' socio-demographic characteristics, most reliable sources of information, percentages on the use of protective devices, and changes in daily life habits are shown in Supplementary Table 2.

### T2 Main Results

Table 4 reports the subjects' mean scores on the global cognitive tasks and their socioeconomic status (SES). Specifically, they performed well on all cognitive tests, without any scores under the cut-off, on ACE-R, MMSE, and MoCA.

**Table 4:** Follow-up: socio-demographic characteristics and neuropsychological assessment (T2).

	***N***	**M**	**SD**	**Cut-off**
**Socio-demographic characteristics**				
Subjects	50			
Gender [male/female]	10/40			
Age [years]		70.04	5.70	
Education [years]		12.84	2.76	
SES (hollingshead index)		41.62	9.14	
Social stratum 66–55		8%		
Social stratum 54–40		48%		
Social stratum 39–30		34%		
Social stratum 29–20		10%		
Social stratum 19–8		0%		
**Neuropsychological assessment**				
Mini-mental state examination		29.44	0.67	≥23.8
Addenbrooke's cognitive examination—revised version		95.04	3.37	≥79 (<75 years old);
				≥60 (>75 years old)
Montreal cognitive assessment		27.02	2.61	≥17.363
**Neuropsychiatric assessment**				
Apathy evaluation scale		8.84	6.25	≤ 14
Beck depression inventory		7.70	6.10	≤ 9
Disinhibition scale		2.88	2.35	≤ 16.9
Hamilton rating scale for anxiety		6.64	5.45	≤ 14
Mania scale		1.78	1.72	≤ 15

*N, number; M, mean; SD, standard deviation. Wherever there is a normative value, the cut-off scores are given in the statistical normal direction*.

On mood assessment scales, they obtained normal scores on DIS-S and MAS; on the other hand, there were under cut-off values on BDI (28%), AES (20%), and HAM-A (10%).

With regard to their SES, according to the Hollingshead Index (HI) 8% of the subjects fell into the highest social stratum, 48% in the second, 34% in the third, 10% in the fourth, and none of them in the lowest one.

### T3 Main Results

The scores obtained at T3 on LFS, BDI, and HAM-A are reported in Table 5.

**Table 5:** Follow-up: psychological assessment (T3).

	**M ± SD** **(*N* = 50)**	**Low-mild fatigue** **(*N* = 25)**	**Moderate-severe fatigue** **(*N* = 25)**	***t*-scores**	***P*-** **values**
LFS	23.18 ± 8.40	16.68 ± 3.40	29.68 ± 6.72		
BDI	8.32 ± 7.24	3.84 ± 2.82	12.80 ± 7.70	5.46071	0.000002
HAM-A	7.86 ± 6.71	3.80 ± 3.30	11.92 ± 6.96	5.27110	0.000003

*M, mean; SD, standard deviation; N, number; LFS, Lockdown Fatigue Scale; BDI, Beck Depression Inventory; HAM-A, Hamilton Rating Scale for anxiety*.

*The subjects were divided into two groups with reference to LFS scores (low-mild and moderate-severe level of fatigue) and a comparison was made on mood changes between the two groups. Significant results are expressed in t-scores and P-values*.

Although results on psychological scales indicated mean scores in the normal range, 28% of subjects showed mood deflections in terms of depression (BDI) and 18% in terms of anxiety (HAM-A).

Regarding LFS scores, 10% of the sample fell into low level of fatigue, 56% into mild level, 30% presented moderate fatigue, while 4% reported severe lockdown fatigue.

In order to test for possible differences concerning the degree of lockdown fatigue, the subjects were divided into two groups according to the median scores: low-mild level of fatigue (17 females and 8 males) and moderate-severe level of fatigue (23 females and 2 males). Interestingly, 62.5% of the pre-frail subjects fell into the moderate-severe fatigue group.

Considering these two groups, the *t*-test showed a significant difference in terms of mood changes. Specifically, subjects feeling more fatigued due to the lockdown presented higher levels of anxiety, in terms of HAM-A scores (*t-value* = 5.27110; *p* = 0.000003), and depression as assessed by BDI (*t-value* = 5.46071; *p* = 0.000002).

On the basis of these findings, we assessed our hypotheses regarding the role of specific cognitive, physical, and psychological aspects in the emergence of lockdown fatigue.

A significant moderated-mediation model (see Supplementary Figure 1) highlighted the interacting effects of psychomotor speed, gait speed, and depression at T0 on lockdown fatigue scale at T3 (see Table 6). Indeed, a conditional process analysis showed that the effect of TMT-A performance on lockdown fatigue at T3 was mediated by depression, but this mediation was moderated by gait speed. Namely, a significant interaction between low psychomotor speed (TMT-A) and low gait speed (i.e., moderation) was associated with stronger mood deflection. Therefore, only at low gait speed the strength of depressive state mediated the enhancing effect of low psychomotor speed on lockdown fatigue.

**Table 6:** Moderated-mediation analysis for predicting lockdown fatigue scale at T3 based on TMT-A, BDI, and gait speed at T0.

**Model summary**						
**Outcome variable: lockdown fatigue scale at T3**						
***R***	***R***^**2**^	**MSE**	***F***	**df1**	**df2**	***p***
0.6297	0.3965	48.4091	7.0476	5	44	0.0001
**Model**						
	**coeff**	**se**	***t***	***p***	**LLCI**	**ULCI**
TMT-A at T0	−0.1549	0.1099	−1.4097	0.1656	−0.3394	0.0297
BDI at T0	0.5074	0.1884	2.6924	0.01	0.1907	0.824
Age	−0.3582	0.2456	−1.4584	0.1518	−0.7708	0.0545
Gender	4.4119	1.9601	2.2508	0.0294	1.1184	7.7054
Educational level	−0.2855	0.34	−0.8396	0.4057	−0.8567	0.2858
**Direct effect of TMT-A at T0 on lockdown fatigue scale at T3**						
	**Effect**	**se**	***t***	***p***	**LLCI**	**ULCI**
TMT-A at T0	−0.1549	0.1099	−1.4097	0.1656	−0.3394	0.0297
**Conditional indirect effects of TMT-A at T0 on lockdown fatigue scale at T3**						
**Indirect effect: TMT-A-T0** **→** **BDI-TO** **→** **Lockdown fatigue-T3**						
Gait speed at T0	**Effect**	**BootSE**	**BootLLCI**	**BootULCI**		
−1 SD	0.0612	0.0603	−0.0441	0.1483		
0	−0.0249	0.0419	−0.1099	0.0232		
+1 SD	−0.111	0.0739	−0.2491	−0.0142		
**Index of moderated mediation**						
**Gait speed at T0**	**Index**	**BootSE**	**BootLLCI**	**BootULCI**		
	−0.0861	0.0529	−0.1755	−0.0076		

*MSE, Mean Squared Error; df, degrees of freedom; LLCI, lower level of confidence interval; ULCI, upper level of confidence interval; coeff, coefficient; se, standard error; SD, standard deviation. Data are reported with coefficients and a 95% confidence intervals*.

## Discussion

To the best of our knowledge, this is the first study in the literature monitoring healthy cognitive aging subjects before and after very restrictive COVID-19 containment measures. We analyzed the impact of the lockdown fatigue in relation to pre-existing aspects of physical frailty, cognitive functioning, and mood deflections. Indeed, T0 data allowed us to better study the associations between neuropsychological variables assessed before the pandemic and the subsequent home confinement.

Although most of the participants were robust at T0, some of them were pre-frail or frail. Only a few minor neuropsychological deficits were observed in cognitive functioning. However, mood changes, particularly in terms of depression, were present in about 40% of the population.

We considered the association among the main characteristics of frailty and cognitive and behavioral aspects in a socially active population with a medium-high SES.

In the first place, the contribution of “cognitive” and “psychological” factors was estimated, resulting from PCAs, in order to explain variability in grip strength and gait speed. The choice of these frailty determinants, associated with cognitive changes, was also supported by studies on community-dwelling people. In particular, Robertson et al. (2014) showed that individuals with slow gait and weak grip had lower scores on executive functions tests. Slow gait speed also showed an important effect on attention and psychomotor speed. Asthenia was associated with global cognition, while low physical activity and unintentional weight loss were not independently associated with cognitive domains. Moreover, a small number of studies on patients with functional and/or cognitive impairment, due to major neurocognitive disorder, highlighted a relationship between a reduction in gait speed or grip strength and impaired attention and executive dysfunction (i.e., Hooghiemstra et al., 2017). Nevertheless, executive-attentional functions mediated by the prefrontal cortex were also related to gait speed in physical frailty (Amboni et al., 2013).

Mood changes—particularly depression and anxiety—are not only common in older people but they are also significant risk factors for frailty. For instance, in a cross-sectional study on community-dwelling older people, Ní Mhaoláin et al. (2012) found that higher levels of depression and anxiety were more prevalent in pre-frail and frail subjects than in robust ones. In addition, they showed a significantly higher probability of anxiety and depressive symptoms. Some authors reported how depression could be considered not only a consequence but also a risk factor for frailty (i.e., Robertson et al., 2013). Particularly, a decrease in handgrip strength was associated with an increased depressive symptomatology (Brooks et al., 2020), which seemed also to be a predictor of slow walking speed (Staples et al., 2020). Another study showed that higher levels of depression and anxiety were associated with a slowdown in gait speed in subjects aged 65 and older with atrial fibrillation (Marino et al., 2019). On the other hand, anxiety seemed to be inversely related to grip strength in the older population (Gordon et al., 2019).

Our results showed how increased grip strength reflects better Attention/Executive performances and lower mood changes, in terms of depression, apathy, and anxiety. In the same direction, a variance in gait speed was also explained by Attention/Executive performance and depression-apathy-anxiety mood changes. Significantly, the contribution of these factors was not explained by age, comorbidity, or educational level.

T1 results showed that almost all subjects took the precautions recommended by the Italian Ministry of Health (Ministero della Salute - Italian Ministry of Health, 2020) to prevent SARS-CoV-2 infection. It is noteworthy that the majority of our sample (90%) belongs to the middle-high social class, according to HI. Combined with a high level of education, such aspect highlights the peculiarity of this population, characterized by social resources, which allowed them to take the necessary precautions to avoid the risk of contagion.

Our T2 results showed no cognitive decline nor mood deflection. However, some under cut-off scores were found in terms of depression, apathy, and anxiety. These aspects align with previous studies on the general population during the COVID-19 pandemic (Prati and Mancini, 2021).

With regard to the subsequent lockdown (T3), which occurred from December 2020 to January 2021, subjects complaining of higher lockdown fatigue exhibited increased levels of depression and anxiety than those with lower fatigue.

These results are consistent with another study on COVID-19 lockdown fatigue (Field et al., 2021), in which the fatigue was related to depression and anxiety but also to sleep disturbance, increased posttraumatic stress symptoms, and a worsened lifestyle (e.g., decreased physical activity, fewer experiences of recreational activities, fewer interactions with others, and more time spent gaming and chatting about the virus). In particular, psychological factors, such as depression, sleep disturbance, and anxiety, explained 51% of the variance of lockdown fatigue (Field et al., 2021).

The mechanism related to physical, behavioral and cognitive factors, and LFS is still unknown. Therefore, we used a moderate-mediation model to explore the complex pathways leading to the onset of lockdown pandemic fatigue. Specifically, we examined the potential mediating effect of depression and attention, and the moderated effect of gait speed in this well-established association. We found a two-way interaction (moderation) between TMT-A and gait speed in influencing the mediator BDI; thus, when walking speed is below the mean, limited psychomotor speed resources are associated with greater depression. Consequently, for above-average slow walk values, the depressive state mediates the effect of limited psychomotor speed resources on subsequent fatigue. Some studies have highlighted how not only attention performances are impaired in normal aging (e.g., Periáñez et al., 2007), but also how usual walking speed is associated with slower test performance on TMT-A in MCI patients (Knapstad et al., 2019). The association between depression and psychomotor speed is already well-known. Patients with major depressive disorders aged 60 years and older showed worse attention abilities, especially when frail (Potter et al., 2016).

Concerning the lockdown pandemic fatigue, higher scores of depression and everyday fatigue were found in the Polish population during COVID-19 home confinement (Bartoszek et al., 2020). Moreover, a previous study on social distancing issues related to the COVID-19 pandemic in the general population (aged 18 years and older) showed an association between exhaustion and depressive symptoms (Seiter and Curran, 2021). Indeed, increased stress levels due to pandemic uncertainty and restrictions could trigger depressive symptoms and greater fatigue burden (Schrack et al., 2020).

A recent study investigated the psychological effects of lockdown on cognitive functions showing general deterioration, in particular regarding concentration and attention (Fiorenzato et al., 2021). Difficulties in keeping focused and concentrated could lead to mental fatigue and, consequently, to greater feelings of lockdown pandemic fatigue in older adults.

### Conclusion

To our knowledge, this is the first study examining the association among specific cognitive, physical, behavioral, and functional characteristics before, during, and after restrictive lockdown measures in cognitively normal aging subjects. It is important to underline how UNITRE participants, with mild neuropsychological and physical alterations, represent an optimal reference sample to implement possible primary prevention pathways on older adults.

Our results highlight that: (a) physical functions, executive attention, and mood changes can play an important role in the current COVID-19 pandemic; (b) the subjects showing moderate-severe fatigue reported more depressive and anxiety issues than subjects with low-mild fatigue; (c) cognitive functioning, in terms of psychomotor speed, seems also to play an important role in the perception of fatigue due to COVID-19 restrictive measures.

Since lockdown fatigue is related to mood deflections, such as depression and anxiety, it would be useful to study this aspect more in depth, as well as other COVID-19 related psychological issues (Field et al., 2021).

### Limitations Section

Although this novel study was carefully designed and achieved its aims, the sample size, set at 50 participants, may be considered a critical aspect.

Furthermore, because of the COVID-19 containment measures in place, it was not possible to perform a proper face-to-face neuropsychogeriatric assessment, as in T0. The restrictions led us to select the most suitable neuropsychological tests for remote administration in line with our aims. To address any potential critical issue related to the remote administration of neuropsychological tests, we adopted *ad hoc* tools for screen sharing in video calls (e.g., MOCA-Blind test).

## Data Availability Statement

The raw data supporting the conclusions of this article will be made available by the authors, without undue reservation.

## Ethics Statement

The studies involving human participants were reviewed and approved by Ethics Committee of the University of Turin. The patients/participants provided their written informed consent to participate in this study.

## Author Contributions

MA conceptualized and designed the study, wrote the draft of the manuscript, reviewed it, supervised the project and was responsible for funding acquisition. NC analyzed study data, wrote the draft of the manuscript, and reviewed it. MB and GEC took part in the writing process, edited the manuscript, and collected data. SP collected data and took part in the writing process. SC reviewed the manuscript and supervised the project. All authors listed have made a substantial contribution to the conception, development of methodological approach and interpretation of results, and approved it for publication.

## Conflict of Interest

The authors declare that the research was conducted in the absence of any commercial or financial relationships that could be construed as a potential conflict of interest.

## Supplementary Material

Supplementary Figure 1 The moderate-mediation model, adapted from Hayes' model 7 (page 588). BDI, Beck Depression Inventory; TMT-A, Trail Making Test- Part A; LFS, Lockdown Fatigue Scale.
