# Supplementary material for: Lockdown Effects on Healthy Cognitive Aging During the COVID-19 Pandemic: A Longitudinal Study
Source: Front Psychol. 2021 May 24;12:685180. doi: 10.3389/fpsyg.2021.685180 (PMC8180921; doi:10.3389/fpsyg.2021.685180)
Supplement: Supplementary file 1 [file Table_1.docx]

**Lockdown effects on healthy cognitive aging during the COVID-19 pandemic: A longitudinal study**

***Supplementary Materials***

**Table S1. Relative frequency of different types of chronic pathologies detected in the global sample at baseline (T0)**

| Pathologies | Frequency | Maximum |
| --- | --- | --- |
| Cardiac | 11/81 | 1 |
| Hypertension | 42/81 | 1 |
| Vascular, hematological | 9/81 | 1 |
| Respiratory | 3/81 | 1 |
| Eye, ear, nose, throat, larynx | 10/81 | 1 |
| Upper gastrointestinal | 6/81 | 1 |
| Lower gastrointestinal | 4/81 | 1 |
| Hepatic | 10/81 | 1 |
| Renal | 3/81 | 1 |
| Genitourinary | 2/81 | 1 |
| Musculoskeletal, integumentary | 28/81 | 2 |
| Neurological | 6/81 | 1 |
| Endocrine, metabolic | 25/81 | 2 |
| Psychiatric/behavioral | 2/81 | 1 |
| Neoplasia | 11/81 | 1 |

*Note*. The table reports the relative frequency of different types of chronic pathologies in our global sample at the baseline and before the lockdown restrictive measures, alongside the maximum number of distinct pathologies belonging to the same category (e.g. endocrine-metabolic) reported by a single participant.

**Table S2. Socio-demographic characteristics, pandemic-related information, and changes in habits of the study population at T1**

|  | N | | % | M | SD | improve-ment | simila-rity | worse-ning |
| --- | --- | --- | --- | --- | --- | --- | --- | --- |
| **Socio-demographic characteristics** | |  | |  |  |  |  |  |
| Subjects | 50 | |  |  |  |  |  |  |
| Gender [M/F] | 10/40 | |  |  |  |  |  |  |
| Age [years] |  | |  | 70.02 | 5.69 |  |  |  |
| Education [years] |  | |  | 12.84 | 2.76 |  |  |  |
| Housing status [single/cohabitant] | 19/31 | |  |  |  |  |  |  |
| **Information pandemic-related** |  | |  |  |  |  |  |  |
| Health conditions |  | |  |  |  |  |  |  |
| symptoms COVID-19 related |  | | 18% |  |  |  |  |  |
| medical examination |  | | 8% |  |  |  |  |  |
| nasal swap |  | | 2% |  |  |  |  |  |
| positivity to COVID-19 |  | | 0% |  |  |  |  |  |
| Main preventive measures |  | |  |  |  |  |  |  |
| using face masks |  | | 96% |  |  |  |  |  |
| wearing latex gloves |  | | 90% |  |  |  |  |  |
| keeping social safety distance:1.5-2 metres |  | | 96% |  |  |  |  |  |
| washing hands (rubbing with soap for at least 60 seconds) |  | | 96% |  |  |  |  |  |
| avoiding crowded places |  | | 84% |  |  |  |  |  |
| Sources of information |  | |  |  |  |  |  |  |
| newscast |  | | 90% |  |  |  |  |  |
| newspaper |  | | 60% |  |  |  |  |  |
| websites and social networks |  | | 6% |  |  |  |  |  |
| television programs |  | | 38% |  |  |  |  |  |
| other |  | | 12% |  |  |  |  |  |
| **Changes in habits** |  | |  |  |  |  |  |  |
| cognitively stimulating activities |  | |  |  |  | 0% | 90% | 10% |
| physical activity |  | |  |  |  | 0% | 30% | 70% |
| diet |  | |  |  |  | 2% | 84% | 12% |
| social interactions |  | |  |  |  | 0% | 6% | 94% |
| sleep |  | |  |  |  | 12% | 56% | 32% |
| smoking, drugs, and alcohol |  | |  |  |  | 0% | 100% | 0% |

*Note:* N= number. M= mean. SD= standard deviation. M= male. F= female.
