# Supplementary figures and images for: Lockdown Effects on Healthy Cognitive Aging During the COVID-19 Pandemic: A Longitudinal Study
Source: Front Psychol. 2021 May 24;12:685180. doi: 10.3389/fpsyg.2021.685180 (PMC8180921; doi:10.3389/fpsyg.2021.685180)

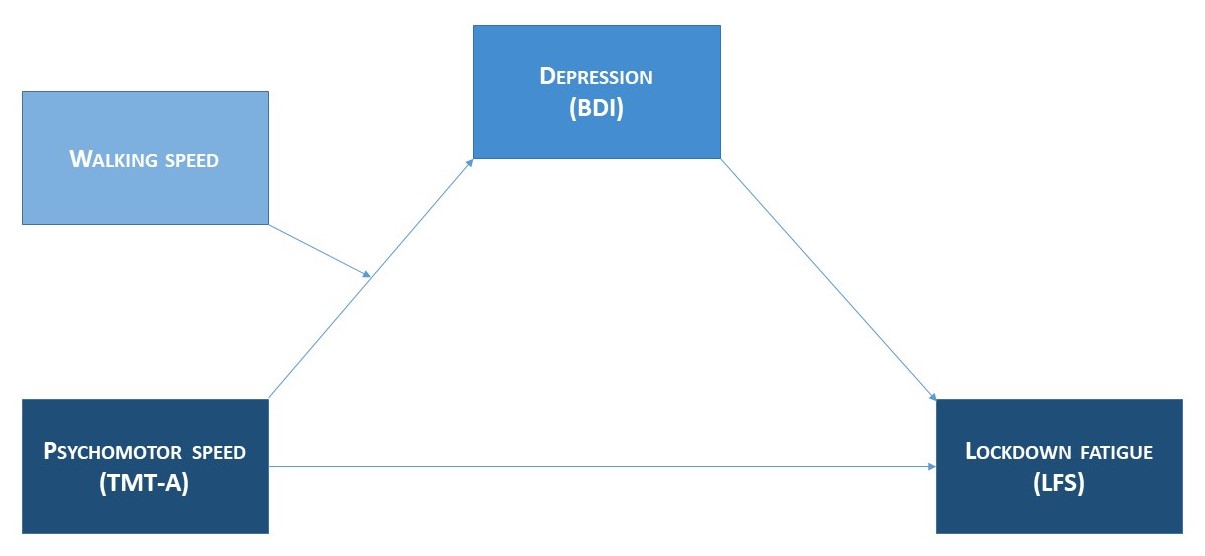

Supplement: Supplementary Figure 1 — The moderate-mediation model, adapted from Hayes' model 7 (page 588). BDI, Beck Depression Inventory; TMT-A, Trail Making Test- Part A; LFS, Lockdown Fatigue Scale. [file Image_1.JPEG]
